# Maternal and Neonatal Directed Assessment of Technologies (MANDATE): Methods and Assumptions for a Predictive Model for Maternal, Fetal, and Neonatal Mortality Interventions

**DOI:** 10.9745/GHSP-D-16-00174

**Published:** 2017-12-28

**Authors:** Bonnie Jones-Hepler, Katelin Moran, Jennifer Griffin, Elizabeth M McClure, Doris Rouse, Carolina Barbosa, Emily MacGuire, Elizabeth Robbins, Robert L Goldenberg

**Affiliations:** a RTI International, Research Triangle Park, NC, USA.; b Department of Obstetrics and Gynecology, Columbia University, New York, NY, USA.

## Abstract

Maternal, fetal, and neonatal mortality disproportionately impact low- and middle-income countries, and many current interventions that can save lives are often not available nor appropriate for these settings. Maternal and Neonatal Directed Assessment of Technologies (MANDATE) is a mathematical model designed to evaluate which interventions have the greatest potential to save maternal, fetal, and neonatal lives saved in sub-Saharan Africa and India. The MANDATE decision-support model includes interventions such as preventive interventions, diagnostics, treatments, and transfers to different care settings to compare the relative impact of different interventions on mortality outcomes. The model is calibrated and validated based on historical and current rates of disease in sub-Saharan Africa and India. In addition, each maternal, fetal, or newborn condition included in MANDATE considers disease rates specific to sub-Saharan Africa and India projected to intervention rates similar to those seen in high-income countries. Limitations include variance in quality of data to inform the estimates and generalizability of findings of the effectiveness of the interventions. The model serves as a valuable resource to compare the potential impact of multiple interventions, which could help reduce maternal, fetal, and neonatal mortality in low-resource settings. The user should be aware of assumptions in evaluating the model and interpret results accordingly.

## Abstract (precis)

MANDATE is a mathematical model designed to estimate the relative impact of different interventions on maternal, fetal, and neonatal lives saved in sub-Saharan Africa and India. A key advantage is that it allows users to explore the contribution of preventive interventions, diagnostics, treatments, and transfers to higher levels of care to mortality reductions, and at different levels of penetration, utilization, and efficacy.

## BACKGROUND

Nearly 98% of all maternal, fetal, and neonatal mortality occurs in low- and middle-income countries (LMICs).^1^^–^^3^ Most maternal, fetal, and neonatal mortality arises from conditions that are preventable or treatable if appropriate care is available.^1^^,^^4^^,^^5^ However, about half of births in LMICs occur outside a health facility, and about half of home births are not attended by a birth attendant.^6^ With only about half of all deliveries occurring in facilities in LMICs, many lifesaving interventions are unavailable to pregnant women.^7^^,^^8^ Even when they are available, existing interventions are often too complex for unskilled workers, and many maternal, fetal, and neonatal problems began before the onset of childbirth. The high skill level required and the lack of infrastructure hinder widespread adoption of many interventions that could reduce maternal, fetal, and neonatal mortality. To address the challenges to safe pregnancy and childbirth in LMICs, innovative solutions are needed.

When researchers assess the impact of a health intervention, it is often based on efficacy in a controlled clinical setting or on the availability of the intervention within LMICs.^9^ In addition, clinical research is expensive, and randomized trials of known efficacious interventions are often difficult to conduct in LMICs. Therefore, knowledge gaps exist between the potential benefit of interventions in controlled clinical trial settings and the potential benefit of realistic maternal, fetal, and neonatal care in LMICs. The context is critical to consider when evaluating which interventions have the greatest potential to save lives in LMICs.

Knowledge gaps exist between the potential benefit of interventions in controlled clinical trial settings and realistic care in low- and middle-income countries.

One way to address these knowledge gaps is by using mathematical models to estimate the impact of interventions in different settings. Important to interpreting model results is understanding the assumptions that inform the model as well as the limitations and uncertainty of model results. Examples of modeling considerations include the variance in quality of data to inform baseline estimates, especially for low-resource settings; the applicability of efficacy for an intervention studied in a hospital versus clinic versus home setting; the difficulties of supply chain or worker skills in LMICs; and the definition of the intervention itself (i.e., whether providing treatment also assumes that the patient was already diagnosed correctly).

Maternal and Neonatal Directed Assessment of Technologies (MANDATE) is a mathematical model that assesses the potential of individual interventions and combinations of interventions to reduce maternal, fetal, and neonatal mortality in sub-Saharan Africa and India. It is a web-based decision-support tool (www.mnhtehc.org) that compares the relative effect of different maternal, fetal, and neonatal interventions and provides insights on potential bottlenecks that might prevent an intervention from saving the maximum number of maternal, fetal, and neonatal lives.^9^^–^^11^ Researchers, universities, technology developers, and ministries of health could potentially use MANDATE as a tool for developing and optimizing their maternal, fetal, and neonatal interventions.

The objective of this paper is to describe the modeling methods used to develop the MANDATE model as well as the data and processes used to calibrate and validate the model. We also discuss the strengths and limitations of the MANDATE model, which may be applicable to other models.

## METHODS FOR DEVELOPING THE MANDATE MODEL

### Source of the Data

To collect information on maternal, fetal, and neonatal conditions and interventions, we conducted a literature review that included all literature on maternal, fetal, and newborn mortality and interventions published in English from 1980 through April 2015 in PubMed, the Cochrane Library, and the World Health Organization database, resulting in the review of 1,401 articles. Specifically, literature was reviewed that addressed maternal, fetal, and neonatal mortality rates and interventions in sub-Saharan Africa and India. Where available, Cochrane reviews were used to establish the efficacy of interventions to reduce maternal, fetal, and neonatal mortality in LMICs. Demographic and Health Surveys data were used to estimate the availability of interventions, and United Nations reports were used to estimate the number of live births per region or country. Incidence and mortality rates from conditions affecting maternal, fetal, and neonatal health were established using journal articles that addressed all-cause mortality in LMICs.^1^^–^^5^^,^^12^^–^^16^

When data were unavailable from these sources, we gathered data through expert opinion, including the Global Network for Women's and Children's Health Research,^17^ a research network in Argentina, the Democratic Republic of the Congo, Guatemala, India, Kenya, Pakistan, and Zambia. Key citations are available for each intervention on the MANDATE website (www.mnhtech.org). Finally, a modified graph decorrelation (GraDe) algorithm was applied, which used the estimates from the highest quality sources as primary, with support from other sources where no other data were available. Key references are denoted beside each intervention on the MANDATE website, and can be seen by clicking the question mark beside each intervention.

### Modeled Conditions

MANDATE evaluates the major conditions that cause maternal, fetal, and neonatal mortality, excluding unsafe abortion, and the impact of a range of interventions to prevent, diagnose, or treat each condition. To develop the model, we first determined the conditions that have the greatest impact on maternal, fetal, and neonatal mortality in sub-Saharan Africa and India. The model includes conditions affecting maternal, fetal, and neonatal mortality and their related sub-conditions, as identified through the WHO International Classification of Diseases.

MANDATE evaluates the major conditions that cause maternal, fetal, and neonatal mortality and the impact of interventions to prevent, diagnose, or treat each condition.

**FIGURE 1.**
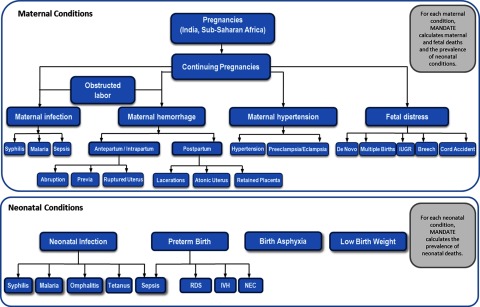
Conditions Modeled in MANDATE Abbreviations: IUGR, intrauterine growth restriction; IVH, intraventricular hemorrhage; NEC, necrotizing enterocolitis; RDS, respiratory distress syndrome.

Modeled conditions associated with maternal mortality include obstructed labor, maternal infection, maternal hemorrhage, and maternal hypertensive disorders. Modeled conditions associated with stillbirth include obstructed labor, maternal hemorrhage, maternal hypertensive disorders, fetal distress, and maternal infections. Modeled neonatal conditions include infection, birth asphyxia, and preterm birth. Within each condition, sub-conditions that are attributed to each cause were also defined (Figure 1). Sub-conditions refer to specific etiologies of conditions; for example, maternal infection, which is the main condition, includes sepsis, syphilis, and malaria as its sub-conditions.

Evaluating each sub-condition allows for interventions to be applied only to the appropriate population that could benefit from the intervention. For example, even though maternal hemorrhage is often discussed as a cause of maternal mortality, hemorrhage is caused by several sub-conditions such as placental abruption, placenta previa, ruptured uterus, lacerations, atonic uterus, or retained placenta. Treatments for these sub-conditions vary. For example, with the antepartum or intrapartum hemorrhages (e.g., placental abruption, placenta previa, and ruptured uterus), clinicians need to consider the status of the fetus (i.e., alive or dead), whereas with postpartum hemorrhages the fetus will not directly benefit from maternal interventions. Similarly, if the cause of a hemorrhage is a retained placenta, suturing the cervix will not treat the underlying cause of the hemorrhage. Interventions within the model are specific to each sub-condition and are only applied to the sub-condition that they impact. The interventions in the model focus on current best practices as well as promising or emerging clinical practices.

After interventions are applied, the maternal sub-conditions are associated with rates of maternal death, fetal death, and the prevalence of a neonatal condition. Neonatal sub-conditions are associated with risk for neonatal mortality and have no impact on maternal or fetal outcomes.

### Mathematical Modeling

**FIGURE 2.**
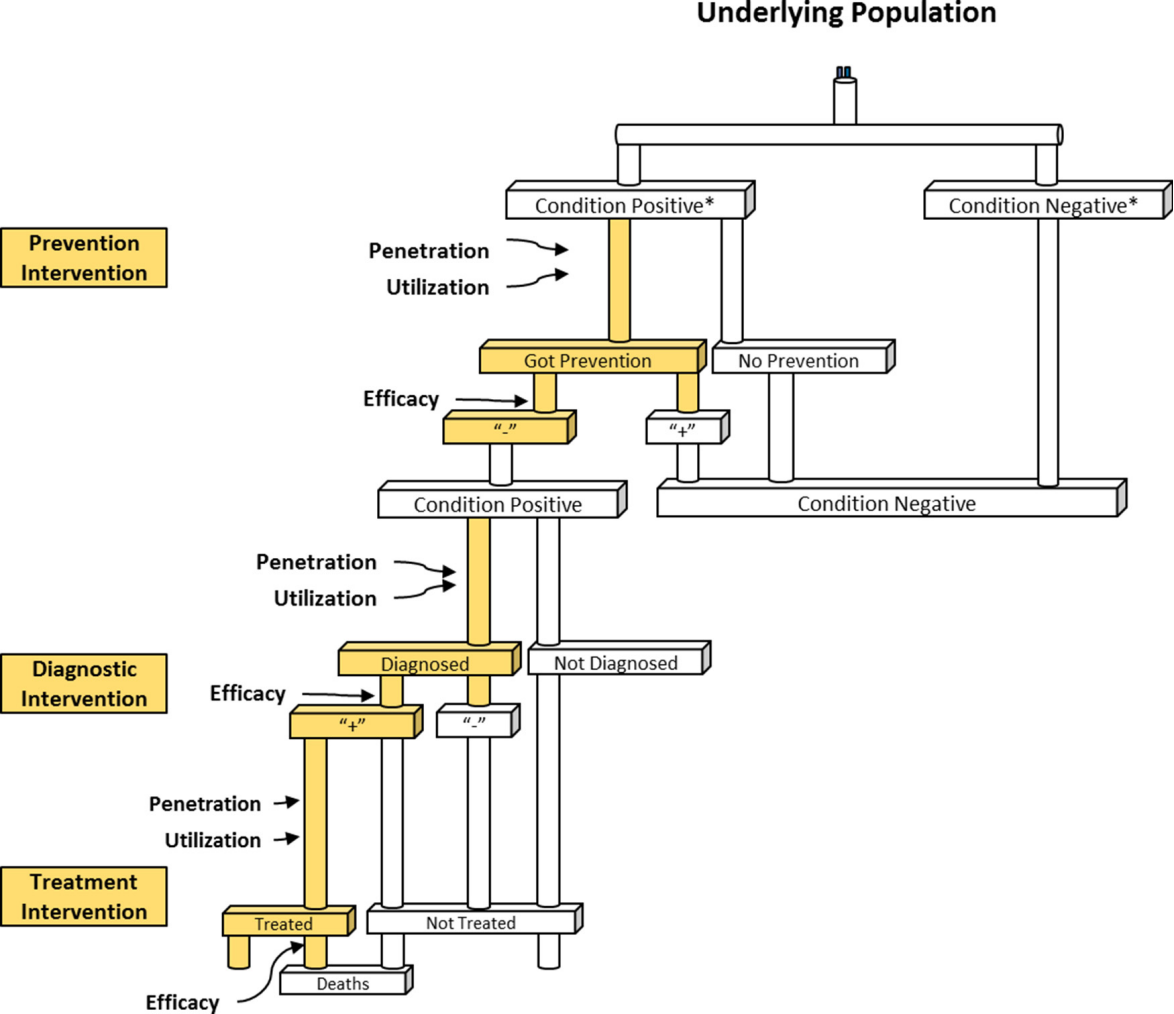
Decision Tree Modeling Methodology *Condition positive and negative is based on an “un-prevented” incidence rate (i.e., the proportion of the population who would get a condition if no preventive interventions were available).

MANDATE is a decision tree mathematical model based on the conditions and sub-conditions (Figure 2). For each of these sub-conditions, the model calculates the number of pregnancies in a particular time frame in the designated geographic region. The model is calibrated using several scenarios. First, the model's initial inputs are calibrated using historical incidence rates (e.g., incidence rates before the existence of antibiotics for the bacterial infection model, or incidence rates before the use of uterotonics or active management of the third stage of labor for hemorrhage) that assume no interventions are available in the population to determine the population at risk of having a sub-condition. Then interventions are added to prevent, diagnose, and treat maternal, fetal, and neonatal sub-conditions using baseline estimates of their availability (i.e., penetration), use when available (i.e., clinically significant/appropriate utilization), and efficacy (i.e., benefit under ideal, controlled conditions). Finally, MANDATE uses untreated case fatality rates to estimate the likelihood of mortality if no interventions are used.

### Interventions

The interventions included in the model to compare their relative impact consist of preventive interventions, diagnostics, treatments, and transfers to different care settings.

#### Preventive Interventions

A preventive intervention is defined as an intervention that reduces the incidence of a sub-condition. In the model, preventive interventions decrease the number of people who develop the sub-condition. Members of the population whose condition was successfully prevented are moved from the pool of individuals with the condition to the condition-negative group. When a condition is successfully prevented, it no longer contributes to the risk of dying from the sub-condition in the model.

#### Diagnostics

MANDATE defines diagnostics as interventions that successfully recognize or diagnose a disease status, assuming the subject is a true positive diagnosis for a particular condition. MANDATE does not allow for false positive diagnoses to receive benefit from treatment, as these individuals in actuality would receive no benefit from the treatment. The model requires a diagnosis of the condition to prompt actions such as treatment or transfer to a facility for treatment. Diagnostics typically fall into 3 categories: (1) recognition of symptoms—made by a patient or unskilled care provider; (2) clinical diagnostics—made by a skilled health care provider; or (3) technology-based diagnostics—technologies used to formally diagnose a condition.

#### Treatments

Treatments are defined as interventions that impact mortality among the sub-conditions; the patient must first be appropriately diagnosed to receive a treatment within the model. A treatment for a mother can also impact fetal sub-conditions or reduce the likelihood of a neonate developing a sub-condition. For example, a cesarean delivery used to treat a mother with preeclampsia may prevent the mother's mortality from preeclampsia and may also prevent fetal mortality and neonatal birth asphyxia.

#### Transfers to Different Care Settings

Preventive interventions, diagnostics, and treatments are each evaluated using 3 constructs: penetration, utilization, and efficacy. Penetration is the availability of an intervention. Utilization is the appropriate use of an intervention. Efficacy is the ability of an intervention to successfully prevent, diagnose, or treat a given sub-condition under ideal conditions. Even though efficacy is defined in the model as constant, regardless of location where the intervention is applied, medical care and availability of interventions often varies based on level of care. These variations are captured by evaluating each intervention option (i.e., preventive interventions, diagnosis, and treatment) in 3 different settings: home, clinic, and hospital.

The variance in medical care and availability of interventions is captured by evaluating each intervention option in 3 different settings: home, clinic, and hospital.

The setting represents where an intervention will occur, which usually corresponds to the location of antenatal or delivery care, or, for conditions with long latency periods, where symptoms emerge. Home settings are defined by having very limited availability of skilled providers, no cesarean or surgical capabilities, and no technology-based interventions. Clinic settings are defined as having some availability of skilled providers who can provide basic obstetric and neonatal care, with no cesarean or invasive surgical capabilities.^18^ Hospital settings are defined as having the availability of skilled providers such as nurses and physicians. Hospitals have varying degrees of emergency obstetric and neonatal care capacity, including cesarean and surgical capabilities in some hospitals.^18^ As such, some interventions are only available or utilized in the clinic or hospital settings. By explicitly modeling the differences in the availability (penetration) and utilization of interventions in home, clinic, and hospital settings, the MANDATE model calculates the differences in mortality by care in different settings.

Transfers are captured in MANDATE as the ability of pregnant women and neonates to move from one setting to another for care. For example, a diagnosis in a home setting might increase the proportion of patients who transfer to a different care setting, such as a clinic or hospital, for additional intervention.

#### Simultaneous Use of Multiple Interventions

The model allows for more than one preventive intervention, diagnostic, or treatment to be available at the same time for any specific sub-condition. In this case, we have assessed whether the interventions can be given independently or if they are dependent (e.g., one intervention must be given before the second intervention). To address the model's ability to assess multiple interventions at once, the MANDATE model uses the modeling concepts of lines and layers.

The model allows for more than one preventive intervention, diagnostic, or treatment to be available at the same time for any specific sub-condition.

The concept of a “line of intervention” refers to any intervention that is given based on previous interventions that were tried and failed (i.e., the sub-condition was not successfully prevented, diagnosed, or treated). For example, if a newborn was given oxygen for respiratory distress syndrome, and oxygen was not sufficient, then a more advanced treatment, such as ventilation, could be given next. Technologies that must be given in a specific order and are dependent on other interventions being offered first are called “lines of intervention.”

Interventions that are not dependent on any other technology are defined as “layered interventions.” In this case, it does not matter if any other preventive intervention, diagnosis, or treatment is being offered at the same time, and the order in which those interventions are modeled is not relevant. An example is the use of bimanual uterine massage and the use of uterotonics, such as oxytocin, to prevent atonic uterus. Using one of these interventions does not preclude the use of the other, nor does the order in which they are used depend on the other.

MANDATE also accounts for interventions that can be given only once or at specific times based on different interventions. For example, a woman who receives a cesarean delivery cannot receive a cesarean delivery twice for the same pregnancy. However, some interventions can be given multiple times and have different benefits based on the timing of the intervention. One example is the use of oxytocin. Oxytocin helps prevent and treat hemorrhage by contracting the uterus. It can be used as a preventive intervention and as a treatment, so using oxytocin twice on the same mother at different points in her care is allowable in MANDATE.

### Validation and Calibration

Every sub-condition within MANDATE was validated and calibrated by running a minimum of 4 scenarios:

1. No intervention based on literature about the natural course of disease or the known course of disease before modern interventions
2. Current-care intervention that reflects current intervention rates in sub-Saharan Africa or India
3. Intervention rates in high-income countries
4. Change(s)-in-care rates for each intervention in the model

The no-intervention scenario uses historical disease rates to evaluate how many women, fetuses, or newborns died from each sub-condition before interventions became available. For example, when calibrating the sepsis models, we used historical disease rates from the early 1900s, before the advent of antibiotics. Similar historical data were used for each sub-condition.

Each model was validated by estimating the number of maternal, fetal, and neonatal deaths resulting from each condition with treatments that are currently available in sub-Saharan Africa and India. Input data for penetration, utilization, and efficacy of each intervention in each care setting were based on estimates of current rates of intervention in sub-Saharan Africa and India. The aggregate outputs result in mortality rates that reflect the current total mortality in sub-Saharan Africa and India, adjusted for mortality causes not included in the model.

Each model was validated by estimating the number of maternal, fetal, and neonatal deaths resulting from each condition with treatments that are currently available in sub-Saharan Africa and India.

Next, the models had data inputs for penetration, utilization, and efficacy in care settings reflective of where care is sought in high-income countries. (Efficacy remained constant because efficacy by definition is the clinical benefit under ideal conditions.) When interventions in MANDATE are improved to the standard of care provided in high-income countries, the mortality for each condition declines to levels consistent with mortality rates in high-income countries.

The final scenarios were specific to each intervention in the model. Each condition was also evaluated using a high estimate and low estimate for expected mortality as well as a high and low estimate for penetration, utilization, and efficacy for each intervention. The results of these scenarios needed to be logical and appropriately scaled when compared with the no-intervention and high-income countries scenarios. These scenarios were validated by experts in the field of maternal, fetal, and neonatal mortality in LMICs.^9^^–^^11^^,^^19^^–^^21^

## DISCUSSION

No model can provide a comprehensive understanding of maternal, fetal, and neonatal mortality alone, and MANDATE is just one of many resources available to analyze interventions in LMIC settings. When assumptions are understood and models are used judiciously, models can provide unique insights to contribute to improvements in maternal, fetal, and neonatal mortality. Therefore, it is important to acknowledge complementary resources available to support critical decisions about maternal, fetal, and neonatal interventions.

One complementary resource is the Lives Saved Tool (LiST), a free software-based tool (part of the Spectrum suite of tools) that estimates mortality averted due to maternal and child health interventions.^19^^,^^20^ Originally funded by the Bill & Melinda Gates Foundation and the United Nations Children's Fund (UNICEF), LiST is widely used by the global maternal and child health community to advocate for needed interventions in LMICs. The model also includes modules on HIV/AIDS and family planning interventions, and has proven itself an important resource for the global health community.

There are several similarities and differences between LiST and MANDATE that are noted in the Table.^20^ While both models contribute to the body of knowledge about how to intervene in LMICs, we believe that any user of a model should understand the underlying assumptions, strengths, and limitations of that model, and use model estimates as a contributing piece of evidence for optimizing interventions in LMICs.

**TABLE.** Similarities and Differences Between LiST and MANDATE

	LiST	MANDATE
**Purpose**	A Microsoft Windows-based software tool used to model the impact of scaling up health interventions aimed at reducing mortality and morbidity in mothers, newborns, and children under 5 years of age	A web-based, mathematical model designed to estimate maternal, fetal, and neonatal lives saved in sub-Saharan Africa and India
**Conditions Included**	Maternal, fetal, newborn, and child health interventions; malaria interventions; and HIV/AIDS interventions	Maternal, fetal, and neonatal health interventions, excluding HIV/AIDS; malaria is only evaluated based on deaths directly attributable to malaria
**Condition Specificity**	Condition level	Condition and sub-condition level
**Intervention Specificity**	Sometimes packages interventions (e.g., active management of the third stage of labor)	Generally unpackages interventions to focus on a specific component of an intervention (e.g., oxytocin, uterine massage, types of diagnostics)
**Intervention Constructs**	Coverage, effectiveness; rates available for some interventions by setting, dependent on topic	Penetration, utilization, efficacy, and transfer between care settings; rates available for each intervention by setting
**Type of Software**	Spectrum software package	Web-based
**Training and Tutorials**	User manual, online tutorials, webinars, and technical assistance	Online 15-minute tutorial and technical assistance
**Cost to Use**	Free	Free
**Outputs**	Number of maternal and child (up to 5 years) deaths, mortality rates/ratios, deaths averted, intermediate outcomes (e.g., stunting, breastfeeding), and single- and multiple-country scenarios	Number of maternal, fetal, and newborn (up to 28 days) deaths, deaths averted, cases averted (e.g., postpartum hemorrhage, eclampsia), and single- and multiple-country scenarios

Abbreviations: LiST, Lives Saved Tool; MANDATE, Maternal and Neonatal Directed Assessment of Technologies.

One of best ways to understand the differences between the 2 models is to consider a case example, such as providing broader coverage of magnesium sulfate (MgSO_4_) in sub-Saharan Africa to treat preeclampsia. Using LiST's model for 2012, we aggregated all countries in sub-Saharan Africa to examine the impact of adding 100% coverage (i.e., similar to MANDATE's availability times penetration) of MgSO_4_ in settings with skilled providers compared with sub-Saharan Africa's baseline coverage of MgSO_4_. LiST assumes that the efficacy of MgSO_4_ prevents mortality, and therefore, MgSO_4_ is a potentially lifesaving treatment. In this scenario, perfect access to and use of MgSO_4_ in settings with skilled providers (i.e., hospitals and clinics) in sub-Saharan Africa would result in approximately 10,000 maternal lives saved in 2012 as estimated by LiST. When the same analysis is done using the current data version of MANDATE (online model version 1.1.81, data version 1.1.122), with MgSO_4_ penetration and utilization of 100% in clinic and hospital settings, MANDATE estimates that only a little over 700 lives would be saved with the addition of MgSO_4_ to all care settings. To understand the differences between the 2 estimates, it is necessary to know the differences in methodologies and assumptions.

Building on a previous MANDATE analysis, we conducted an abbreviated series of analyses using the current data version of MANDATE to understand better the difference in estimated lives saved between the 2 models with the use of MgSO_4_.^21^ The first critical difference is to understand that in MANDATE, the assumption is that MgSO_4_ does not directly prevent mortality, whereas with LiST the efficacy applied to MgSO_4_ is to prevent mortality. Instead, MANDATE models the efficacy of MgSO_4_ to prevent seizures and recurrent seizures. Another critical difference is that MANDATE does not assume diagnosis is available to each MgSO_4_ recipient. While we understand that MgSO_4_ would not be given if a diagnosis had not been made, we also feel that diagnosis is often a complex step toward getting appropriate treatment, and it cannot be taken for granted as a step toward providing safer pregnancies and childbirth in LMICs. If we assume that diagnosis were available to and used (e.g., provided) by every pregnant woman and MgSO_4_ was given when appropriate, MANDATE estimates that approximately 7,500 maternal lives would be saved. Building on this analysis, we could also use MANDATE to estimate lives saved with the assumption that all women were diagnosed and all women were given access to a cesarean delivery or induction if they are given MgSO_4_, and that all women in need of MgSO_4_ receive it. In this scenario, MANDATE estimates that approximately 10,000 maternal lives would be saved. Finally, we could consider a scenario where all women were diagnosed, transferred to an appropriate care setting, provided MgSO_4_ and cesarean delivery or induction, and then we would see mortality decline from approximately 17,000 maternal deaths per year to approximately 5,000 deaths per year, which means that just over 12,000 maternal lives would be saved by this scenario.

This example illustrates some of the important differences between the LiST and MANDATE approaches (e.g., diagnosis assumptions and transfer assumptions), and highlights the importance of understanding the underlying assumptions used for any modeling. Both models start with similar assumptions regarding the population of pregnancies (using the United Nations data).

LiST allows the user to scale up coverage of the known intervention (in this case MgSO_4_) with use in currently acceptable settings (e.g., health facilities with skilled providers). The LiST model with MgSO_4_ assumes that everyone who gets MgSO_4_ and has preeclampsia/eclampsia could be diagnosed, and that MgSO_4_ (rather than induction or cesarean delivery) prevents death directly, regardless of whether the mother receives an emergent or emergency delivery. Thus, even though LiST allows the user to estimate the impact of scaling up the use of MgSO_4_, LiST also assumes the supporting interventions (e.g., diagnoses and treatment options) are available. In doing this, the assessment of the impact of MgSO_4_ by LiST on lives saved focuses on the overall outcome of MgSO_4_ and assumes that the complex steps needed to appropriately manage preeclampsia/eclampsia are available.

MANDATE allows the user to further explore each of the components that contribute to reduction in mortality (e.g., diagnostics, facility care, access to cesarean delivery and labor induction, and transfers, in addition to MgSO_4_) and to explore each of these at different levels of penetration, utilization, and efficacy.

MANDATE allows the user to explore each component that contributes to reduction in mortality at different levels of penetration, utilization, and efficacy.

We see that when all of the coverage is provided, MANDATE and LiST have similar reductions in maternal mortality, and both provide valuable insights to the lifesaving abilities of MgSO_4_. However, MANDATE also highlights the important interactions between MgSO_4_ and other needed steps for maternal care, including diagnosis, transfer between care settings, and treatment capabilities.

### Limitations of MANDATE

As with all models, MANDATE has a defined scope and includes simplifying assumptions. Limitations relate to either scope or data availability. The scope of MANDATE is limited to 2 geographic regions, sub-Saharan Africa and India. The base assumptions regarding a condition's incidence and intervention penetration and utilization are at the continent level for sub-Saharan Africa and at the country level for India. These numbers can be modified by the user if more accurate information or country-specific data are available; however, literature does not currently provide sufficient data to support country-specific data for every modeling assumption. Though incidence, penetration, utilization, and efficacy are not reflective of each country's specific conditions, the proportion of the population flowing through the model may be restricted to the country level in sub-Saharan Africa (e.g., Ethiopia) and state level in India (e.g., Uttar Pradesh). In addition, a user has the ability to modify assumptions online as long as they disclose the rationale for their modifications.

Data availability and data concordance were a challenge for the MANDATE model. Although we searched the peer-reviewed literature, gray literature, and data from sub-Saharan Africa and India, substantial gaps in the data remain. These gaps reflect the relative scarcity of data regarding disease burden and interventions in LMICs. Further, MANDATE is not a stochastic model and does not account for random variation in population flows through the decision tree; this limitation is primarily due to the lack of data to appropriately calibrate a stochastic model. In addition, some conditions may appear to result in rates of mortality that are lower than rates reported by some experts in that field; however, estimates are calibrated based on global estimates of mortality that consider all causes of mortality together.^1^^,^^2^^,^^4^^,^^14^^–^^16^

We attempted to mitigate data availability issues by using expert clinical opinions and data from RTI International, gathered through the Global Network for Women's and Children's Health Research.^17^ Further, we sought to be transparent about the data availability by embedding critical references for each intervention within the online model. In addition, the model allows a user to change the baseline estimates if they are privy to more accurate local data estimates.

MANDATE also requires rigorous data maintenance to ensure updated estimates for penetration, utilization, and efficacy, and without support to update data, the model will be obsolete within a few years.

MANDATE is currently limited to sub-conditions that directly account for the predominance of maternal, fetal, and neonatal mortality. Comorbidities are not modeled in MANDATE, including HIV/AIDS, which is known to have important impacts on many of the modeled conditions. The technologies included in MANDATE are limited to those that (1) focus on preventing maternal, fetal, and neonatal mortality, and (2) are currently part of clinical standard of care or are highly visible, promising interventions.

Finally, we would like to emphasize that the lessons derived from modeling are limited by the extent to which the user understands, accounts for, and respects the limitations of the underlying modeling assumptions. There is always a risk that inappropriate use of a model can result in the over- or underestimation of the potential impact of an intervention. However, when used thoughtfully, models serve as a useful tool to guide conversations, thoughts, or advocacy around specific interventions.

## CONCLUSIONS

MANDATE is the only model that evaluates maternal, fetal, and neonatal conditions and sub-conditions resulting in mortality. Further, MANDATE considers care settings, transfers to facilities for further interventions, and the impact of maternal conditions on fetal and neonatal outcomes. MANDATE is an important decision-making model that the global maternal and child health community can use to assess the relative impact of interventions on maternal, fetal, and neonatal mortality. In countries with limited resources, it is critical to identify and pursue interventions that can most effectively prevent maternal, fetal, and neonatal mortality. MANDATE can serve as a resource to determine the relative benefit of many potential interventions for maternal, fetal, and neonatal mortality in sub-Saharan Africa and India.

MANDATE is the only model that evaluates maternal, fetal, and neonatal conditions and sub-conditions resulting in mortality.

## Notes

**Peer Reviewed**

**Funding:** Bill & Melinda Gates Foundation.
